# Inhibition of p38-MAPK signaling pathway attenuates breast cancer induced bone pain and disease progression in a murine model of cancer-induced bone pain

**DOI:** 10.1186/1744-8069-7-81

**Published:** 2011-10-20

**Authors:** Devki Sukhtankar, Alec Okun, Anupama Chandramouli, Mark A Nelson, Todd W Vanderah, Anne E Cress, Frank Porreca, Tamara King

**Affiliations:** 1 Department of Pharmacology, College of Medicine, University of Arizona, Tucson, AZ 85724, USA; 2 Arizona Cancer Center, University of Arizona, Tucson, AZ 85724, USA; 3 Department of Pathology, College of Medicine, University of Arizona, Tucson, AZ 85724, USA; 4 Department of Cell Biology and Anatomy, University of Arizona, Tucson, AZ 85724, USA

## Abstract

**Background:**

Mechanisms driving cancer-induced bone pain are poorly understood. A central factor implicated to be a key player in the process of tumorigenesis, osteoclastogenesis and nociception is p38 MAPK. We determined the role of p38 MAPK in a mouse model of breast cancer induced bone pain in which mixed osteolytic and osteoblastic remodeling occurs.

**Results:**

In cancer-treated mice, acute as well as chronic inhibition of p38 MAPK with SB203580 blocked flinching and guarding behaviors in a dose-dependent manner whereas no effect on thresholds to tactile stimuli was observed. Radiographic analyses of bones demonstrated that chronic inhibition of p38 MAPK reduced bone loss and incidence of spontaneous fracture in cancer-treated mice. Histological analysis of bones collected from mice treated with the p38 MAPK inhibitor showed complete absence of osteoblastic growth in the intramedullary space as well as significantly reduced tumor burden.

**Conclusions:**

Blockade of non-evoked pain behaviors but not hypersensitivity suggests differences in the underlying mechanisms of specific components of the pain syndrome and a possibility to individualize aspects of pain management. While it is not known whether the role of p38 MAPK signaling can be expanded to other cancers, the data suggest a need for understanding molecular mechanisms and cellular events that initiate and maintain cancer-induced bone pain for effective management for both ongoing pain as well as breakthrough pain.

## Background

Chronic pain is experienced by 30-50% of all cancer patients irrespective of the stage and 70-90% of those with advanced metastatic disease [1,2]. Cancer pain is multifaceted with varying degrees of intensity, at multiple anatomical locations, and characterized by multiple pain descriptors including inflammatory, neuropathic and mechanical that likely have different underlying mechanisms [3,4]. The most commonly diagnosed cancers such as those of lung, prostate, and breast often metastasize to the bone [5,6] and are associated with bone remodeling and eventual bone fractures that contribute to incapacitating pain and limited or total loss of mobility and daily activity. Even though pain is the most frequent and disruptive symptom of the disease, mechanisms that initiate and maintain cancer-induced pain are not clearly understood. This ultimately results in poor pain management as the analgesic treatments (e.g. opiates, bisphosphonates) currently used to treat cancer-induced bone pain are given across prolonged periods of time and are associated with debilitating side effects [7,8] Overall, pain management still remains inadequate in approximately 40% of cancer patients [9,10]. As advances in cancer therapeutics have drastically increased life expectancy of patients, including those with bone metastases, these patients continue to experience pain that can be severe and unpredictable, greatly limiting daily activity resulting in poor quality of life [11,12]. This highlights the need for effective treatment that can be given over long periods of time without development of debilitating side effects associated with currently available treatments.

One potential mediator of tumor-induced bone pain is p38 MAPK, a serine/threonine mitogen activated protein kinase activated by its phosphorylation in various signaling cascades [13-15]. The p38 MAPK signaling pathway has been implicated in mediating inflammatory and neuropathic pain, both of which are thought to contribute to cancer-induced bone pain [16-18]. Moreover, p38 MAPK has also been demonstrated to be important in maturation and synthesis of osteoclasts, cells responsible for osteolysis [19,20]. The p38 MAPK cascade is known to be activated by tumor-induced cellular stress such as inflammatory cytokines [21]. In addition, it is also established as a key player in the induction of cellular effects of cytokines such as Tumor Necrosis Factor α (TNFα) and Interleukin-1β [22-25]. The growing tumor burden, acidic tumor microenvironment, an array of inflammatory mediators produced by the tumor and tumor associated cells in the bone microenvironment and tumor-induced bone degradation all contribute to bone pain [26]. As p38 MAPK plays a key role in each of these variables, we hypothesized that blockade of p38-MAPK signaling might attenuate cancer-induced bone pain and bone loss. The present study determined the role of p38 MAPK in tumor growth, tumor-induced bone loss, and components of tumor-induced bone pain in a mouse model of cancer-induced bone pain in which mammary adenocarcinoma cells were injected and sealed into the intramedullary space of the femur of female mice to allow controlled progression of disease within the bone.

## Results

### Implantation of breast cancer cells 66.1 into the intramedullary space of the femur induces pain-like behaviors in mice

**Figure 1:**
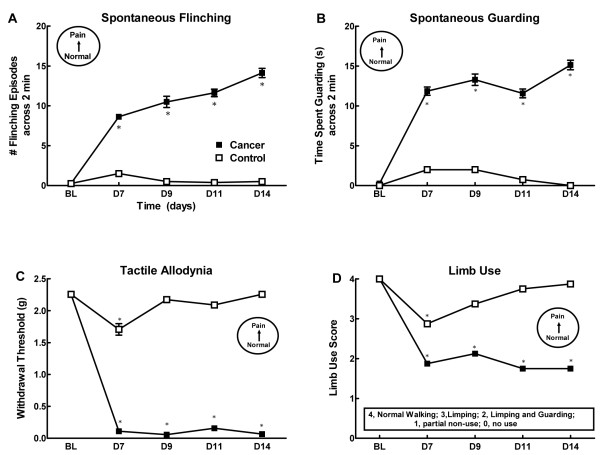
**Time course for emergence of pain behaviors showing spontaneous flinching (A), spontaneous guarding (B), tactile allodynia (C) and limb use score (D)**. n = 8 mice/group.

Injection of the mouse breast cancer cells into the femur produced a time-dependent expression of both non-evoked pain-like behaviors (flinching and guarding) as well as changes in sensory thresholds to evoked stimuli (Figure 1). Cancer-treated mice demonstrated flinching behavior within 7 days post-surgery, with flinching behavior continuing to increase through the day 14 time-point. (Figure 1A). Cancer-treated mice showed significant guarding behavior within 7 days post-surgery, which increased by day 13 (Figure 1B). Cancer-treated mice showed robust tactile hypersensitivity by the day 7 time-point, with paw withdrawal thresholds remaining low throughout the testing period (Figure 1C). Control mice showed slightly lowered withdrawal thresholds at day 7 post-surgery, with withdrawal thresholds returning to baseline by the day 9 time-point. Both cancer-treated and control mice showed impaired limb use on day 7 following the surgery (Figure 1D). Cancer-treated mice showed greater impairment of limb use than control mice, demonstrating both limping and guarding behaviors whereas control mice only showed transient limping behaviors, likely due to surgery induced pain. Impaired limb use was not observed at later time points in control mice whereas cancer-treated mice continued to guard and/or limp through day 14 post-surgery (Figure 1D).

### Implantation of breast cancer cells 66.1 into the intramedullary space of femur induces bone remodeling and tumor growth

**Figure 2:**
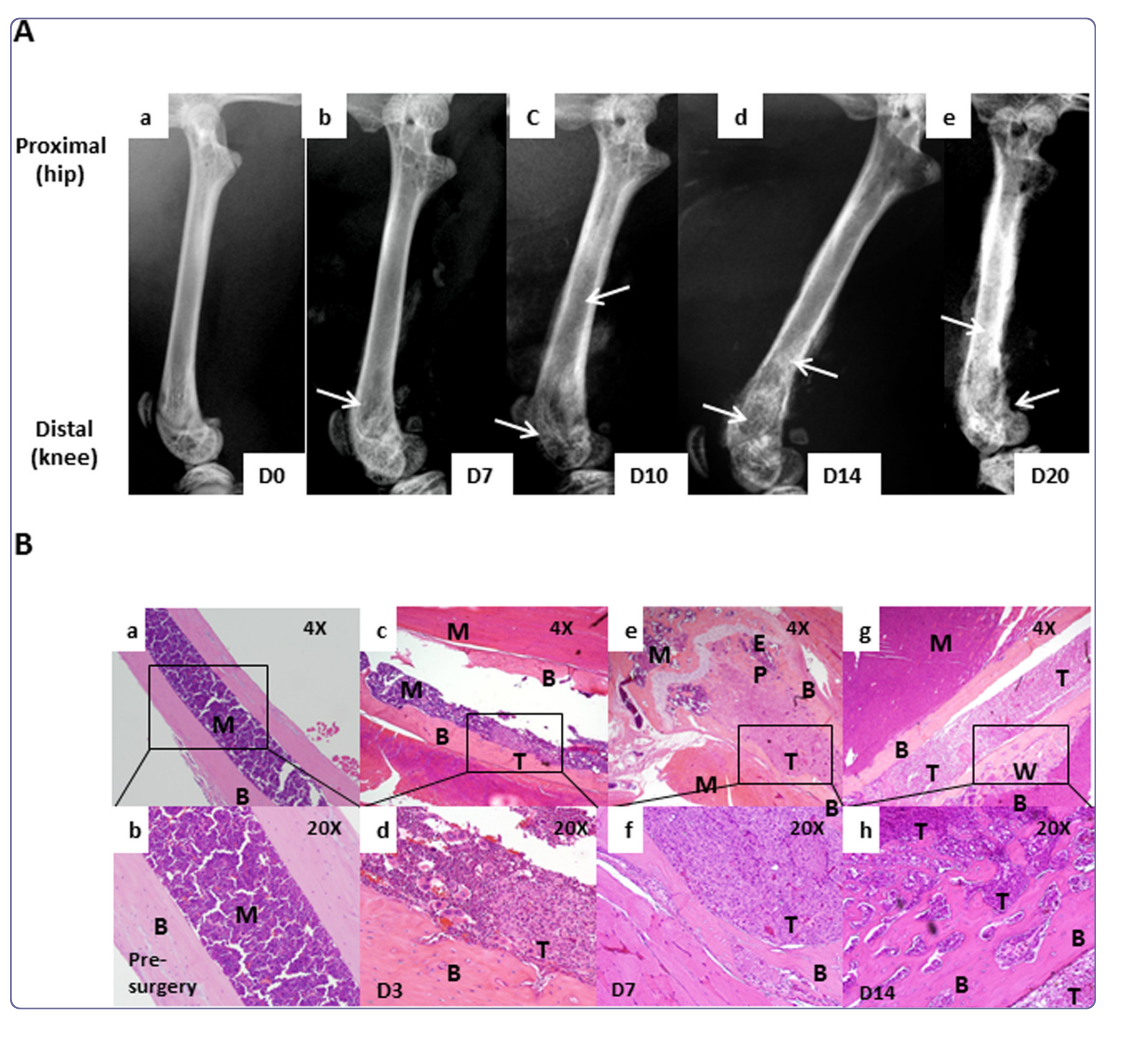
**Breast cancer cells when injected in the intramedullary space of the femur result into disease progression and tumor-induced bone remodeling (A)**. Radiographic images show normal bone with no bone destruction (a), D7 post tumor implantation with little bone loss (osteolytic remodeling; indicated by yellow arrows) observed in the shaft (b), D10 post tumor implantation with increased bone loss in the shaft and knee area (c), D13 post tumor implantation with further increased bone loss in the shaft and the knee region, cortical fractures (indicated by blue arrows) and abnormal bone growth in the shaft (osteoblastic remodeling, indicated by white arrows) (d), Histological analyses of bones (B) show control bone with intact cortical bone and marrow components (a, b), presence of tumor in the intramedullary space D3 post implantation (c, d), tumor invading into cortical bone and growing towards the epiphyseal plate of the knee by D7 post implantation (e,f), tumor infiltrating the entire intramedullary space of the bone with woven bone formation along the cortex by D13(g,h); B, cortical bone; M, bone marrow; T, tumor; EP, epiphyseal plate; WB, woven bone.

Femur radiographs showed a time-dependent change in bone remodeling on days 7, 10, and 13 after the cancer cells were injected (Figure 2A). In comparison with the normal bone (Figure 2A, panel a), cancer-induced bone remodeling was first evident on day 7 post-surgery (Figure 2A, panel b) with bone loss at the proximal end of the bone (knee) at the epiphyseal plate region. Bone loss increased and was extended to the shaft by day 10 post-surgery (Figure 2A, panel c). On day 13 post-surgery, in addition to the bone loss, abnormal bone growth was observed within the shaft (Figure 2A, panel d). Histological analyses of the bones show a time-dependent growth of the tumor within the bone. Bones injected with cell-free media showed normal marrow and continuous cortical bone (Figure 2B, panels a,b). Bones collected on day 3 post-surgery showed the presence of tumor mass within the shaft at the site of injection (Figure 2B, panels c,d). By day 7, tumor burden increased and filled the bone shaft replacing the marrow. In addition, cortical invasion by the tumor was also observed (Figure 2B, panels e,f). On day 13, abnormal osteoblastic bone growth was seen in the shaft as well as growing outside of the cortical bone (Figure 2B, panels g,h).

### Acute administration of SB203580 attenuates breast cancer induced non-evoked pain behaviors

**Figure 3:**
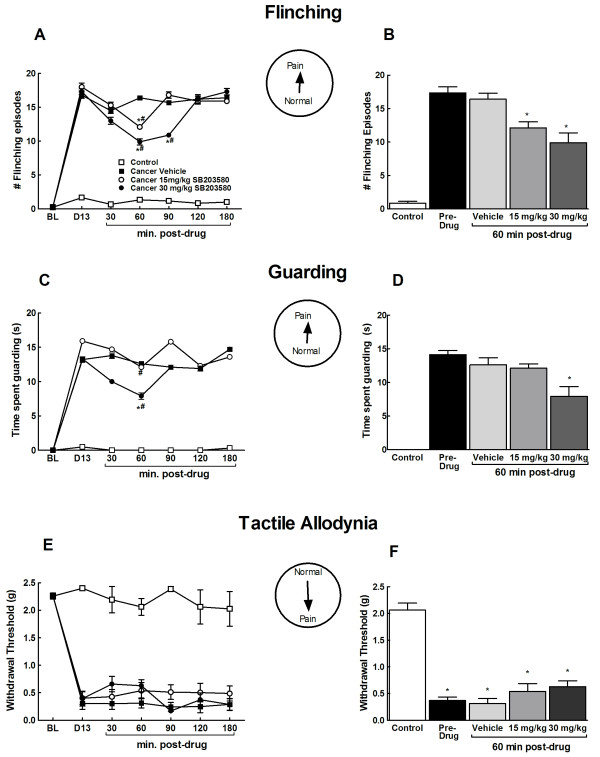
**Effects of acute administration of SB203580 (15 and 30 mg/kg, i.p.) at day 13 post-surgery on cancer-induced spontaneous flinching (A, B), spontaneous guarding (C,D) and tactile allodynia (E,F)**. Time-course analyses indicate a time-dependent decrease in spontaneous flinching (A) and guarding (C) but not tactile allodynia (E).^#^indicates significant difference from pre-drug levels, *indicates significant difference from the vehicle treated group (p < 0.05). Analysis of the peak effect (60 min post-administration, indicates decreased tumor-induced flinching at both doses (B), decreased time spent guarding at the highest dose (D) and no change in tactile hypersensitivity (F). *indicates significant difference from the vehicle treated group (p < 0.05). All graphs show means ± SEM, n = 10.

Pre-drug testing demonstrated that all cancer treated mice showed significant flinching and guarding behaviors 13 days post-surgery whereas control mice did not show any flinching or guarding behavior (Figure 3A-D). SB203580, a p38 MAPK inhibitor, was administered at 15 or 30 mg/kg, i.p. and flinching episodes (Figure 3A,B) as well as time spent guarding (Figure 3C,D) were evaluated across a 2 min period. SB203580 inhibited flinching and guarding with peak drug effects were observed at 60 min post administration (Figure 3A). Flinching and guarding behaviors returned to pre-drug levels by 180 min post administration. Acute systemic administration of p38 MAPK inhibitor decreased flinching in a dose-dependent manner (Figure 3B). Time spent guarding the cancer bearing limb was significantly reduced in mice that received the highest dose (30 mg/kg) of the drug (Figure 3D). Statistical comparison with the pre-drug pain behaviors as well as the vehicle group indicated that flinching was significantly reduced in drug-treated mice at both doses but time spent guarding was only reduced in the mice that received the highest dose (30 mg/kg). Administration of saline vehicle did not alter flinching or guarding in the cancer treated mice.

### Acute administration of SB203580 does not block breast cancer induced tactile hypersensitivity

Pre-drug testing demonstrated that cancer treated mice had decreased response thresholds to von Frey filaments (Figure 3E,F). Acute administration of either dose (15 or 30 mg/kg, i.p.) of the SB203580 failed to diminish tactile hypersensitivity of cancer bearing limb (Figure 3E,F). Paw withdrawal thresholds of mice that received the vehicle did not alter from the pre-drug threshold. Paw withdrawal thresholds of the control mice remained unchanged after the drug administration.

### Chronic administration of SB203580 attenuates breast cancer induced flinching and guarding

**Figure 4:**
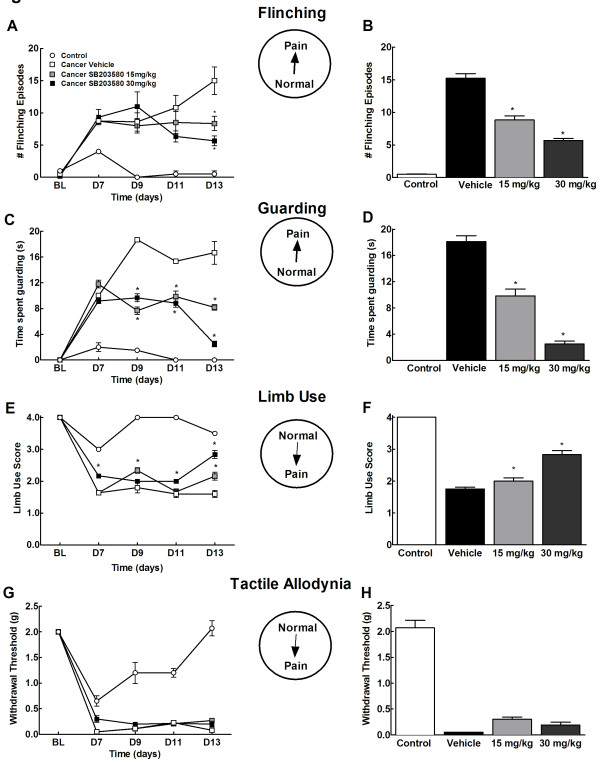
**Effects of chronic administration of SB203580 (15 and 30 mg/kg, i.p., 2× daily) on cancer-induced spontaneous flinching (A,B) spontaneous guarding (C,D), tactile allodynia (E,F) and limb use (G,H)**. Time-course analysis indicate a time-dependent decrease in tumor-induced spontaneous flinching (A) guarding (B) and limb use (E), but no change in tactile hypersensitivity (G). *indicates significant difference from vehicle treated group. Analysis on day 13 demonstrates decreased flinching (B), guarding (D) and limb use (F) in cancer mice treated with 15 as well as 30 mg/kg SB2035580. Chronic treatment with SB203580 failed to alter tactile hypersensitivity at either dose. *indicates significant difference from cancer vehicle group (p < 0.05). All graphs show means ± SEM. n = 6-8.

Before the drug treatment was started (D7), all mice showed flinching and guarding pain behaviors as compared to the controls (Figure 4A,C). Administration of SB203580 (15 and 30 mg/kg, i.p. 2× daily across 7 days starting day 7 post surgery) diminished cancer-induced flinching episodes by D11 post-surgery as compared to those that received vehicle (Figure 4A). By D13, flinching was significantly attenuated in cancer bearing mice treated with the drug (15 mg/kg and 30 mg/kg) compared to those that received vehicle (Figure 4A,B). Cancer bearing mice that received the drug showed reduced time spent guarding the cancer bearing limb by D9 post-surgery and remained diminished throughout the time course in mice that received the drug as compared to those that received the vehicle (Figure 4C,D). Control mice showed minimal flinching and no guarding irrespective of drug treatment on all the test days. Control or cancer treated mice did not show any adverse drug-related effects on prolonged administration of SB203580.

### Chronic administration of SB203580 does not alter breast cancer induced tactile hypersensitivity

Prior to drug treatment (Day 7), cancer-treated mice demonstrated tactile hypersensitivity, as indicated by lowered paw withdrawal thresholds to von Frey filaments. Prolonged treatment with SB203580 (15 and 30 mg/kg) did not alter cancer-induced tactile hypersensitivity across the entire testing period (p > 0.05, Figure 4E,F). Paw withdrawal latencies remained consistent in control mice throughout the testing period irrespective of drug treatment.

### Chronic administration of SB203580 attenuates breast cancer induced impaired limb use

Cancer bearing mice showed impaired limb use by day 7 post surgery as compared to the control mice which showed slight limping behaviors while walking (Figure 4G). By day 13 post surgery, significant improvement in the limb use rating was observed by day 13 post surgery in mice that received 15 as well as 30 mg/kg of SB203580 as compared to vehicle treated mice (Figure 4d). Control mice regained normal limb use by day 9 and maintained throughout the testing period irrespective of drug treatment.

### Chronic administration of SB203580 prevents and/or delays breast cancer induced bone remodeling

**Figure 5:**
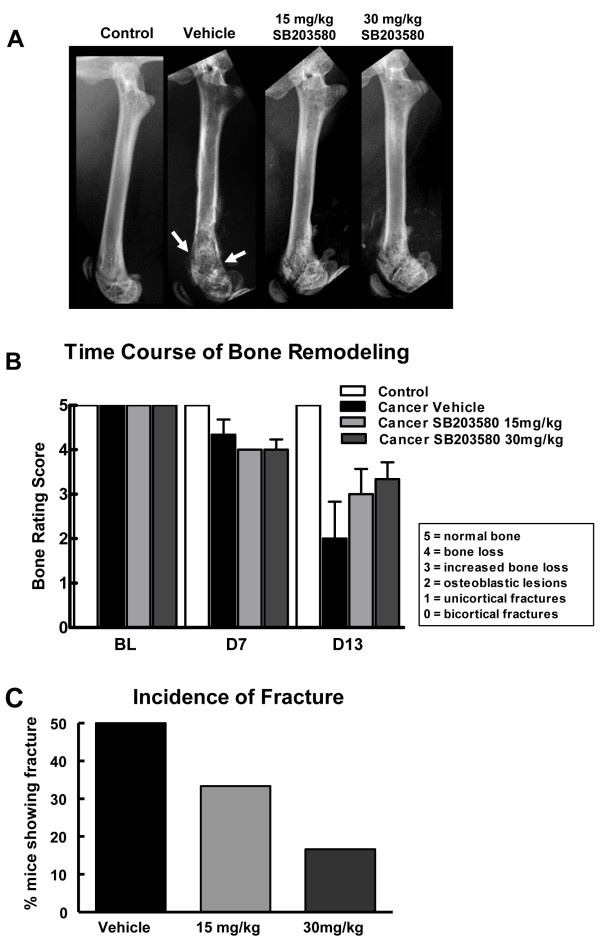
**(A) Radiograph images of the injected femur on day 13**. Breast cancer-induced bone remodeling is reduced in mice that received systemic administration of SB203580 (15 and 30 mg/kg, i.p., 2× daily, 7 days). Fractures as indicated by arrows were defined as full-thickness cortical loss. (B) Bone remodeling ratings of all treatment groups show that the breast cancer-induced bone remodeling is observed 7 days following the surgery, with no difference between drug- and vehicle-treated mice. Breast cancer-treated mice receiving SB203580 (15 and 30 mg/kg, i.p. 2× daily, 7 days) demonstrated reduced bone destruction on day 13 compared with breast cancer-treated mice that received saline. (C) Treatment with SB203580 reduced the percentage of mice with breast cancer-induced spontaneous fractures compared to vehicle-treated animals. n = 6-8.

Mice that received the vehicle demonstrated significant bone loss at the epiphyseal region extending to the shaft of the bone. An abnormal osteoblastic bone growth was also observed at the epiphyseal plate and shaft (Figure 5A). As many as 50% of mice in this group showed unicortical or bicortical fractures (Figure 5B). In mice that received 15 mg/kg or 30 mg/kg of the drug, bone loss at both the regions was greatly reduced, with no evidence of abnormal bone growth (Figure 5A). In addition, prolonged SB203580 treatment reduced incidence of cortical fractures in a dose dependent manner, with only 30% (15 mg/kg) and 15% (30 mg/kg) of the cancer treated mice showing cortical fractures (Figure 5C). This indicates that prolonged inhibition of p38 MAPK diminished bone remodeling. Control mice did not show any abnormal bone remodeling upon drug treatment.

### Chronic administration of SB203580 reduces tumor growth within the intramedullary space of the bone and in vitro

**Figure 6:**
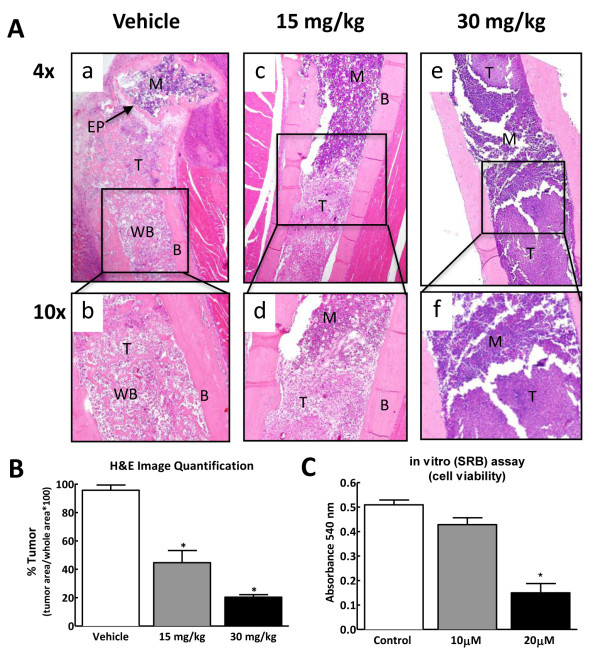
**(A) Representative images of H&E staining showing the effects of chronic p38 MAPK inhibition on cancer treated bones**. Panels a and b demonstrate that breast cancer cells filled the intramedullary space of the femur within 13 days following injection. In addition, areas of new bone growth were evident within the intramedullary space in the breast cancer-treated mice treated with vehicle. The cortical bone displayed an uneven look showing tumor invasion into the bone. Panels c - f show that systemic delivery of the p38 MAPK inhibitor (15 and 30 mg/kg, i.p., 2× daily) across 7 days diminished tumor burden, blocked the new bone growth within the intramedullary space and reduced the invasion of breast cancer cells into the cortical bone. B, cortical bone; M, bone marrow; T, tumor; EP, epiphyseal plate; WB, woven bone. (B) Quantification of tumor bearing area show two fold reduction in mice treated with 15 mg/kg and five-fold reduction in mice treated with 30 mg/kg of SB203580 as compared to vehicle treated mice (n = 4-5). Graph shows mean ± SEM. *indicates significant difference from vehicle-treated bones (p < 0.05) (C) Sulforhodamine B (SRB) assay shows dose dependent reduction in cell viability on treatment of breast cancer cells with SB203580 for 48 h as compared to the breast cancer cells treated with vehicle alone (n = 3). Graph shows means ± SEM. *Indicates significant difference from DMSO-treated (control) group (p < 0.05).

Bones collected from control mice showed apparently normal cortical bone and marrow components. Cancer-treated mice treated with vehicle showed complete infiltration of the marrow by the growing tumor (Figure 6A, panels a,b). Consistency of cortical bone was lost with multiple fractures and abnormal osteoblastic growth throughout the shaft induced by the growing tumor (Figure 6A, panels a,b). In cancer- treated mice that received 15 mg/kg of the drug, tumor growth was confined to the shaft in the middle of the intramedullary space where the breast cancer cells were initially implanted. No tumor was evident around the epiphyseal plate indicating the failure of the cancer cells to migrate with SB203580 treatment (Figure 6A panels c,d). In mice that received 30 mg/kg of the drug, only a few tumor pockets were observed within the intramedullary space. Additionally normal marrow components were still present within the bone (Figure 6A panels e,f). Quantification of the tumor-occupied area in the intramedullary space of the bone indicate a two and five fold reduction of tumor growth in mice treated with 15 or 30 mg/kg SB203580, respectively (Figure 6B). This suggests that prolonged treatment with SB203580 significantly reduced tumor burden within the bone. To determine the effects of p38 MAPK inhibition on 66.1 cell growth, an in vitro sulforhodamine-B assay was carried out. On treatment with SB203580 for 2 days at 20 μM concentration, the number of viable cells was significantly reduced indicating that p38 MAPK is an important determinant of the growth of 66.1 cells and its blockade results into decreased cell number (Figure 6C).

## Discussion

Our data show that (a) acute and prolonged inhibition of p38 MAPK blocks non-evoked pain behaviors as measured by flinching and guarding, but not response thresholds to evoked stimuli; (b) prolonged blockade of p38 MAPK attenuates tumor-induced bone remodeling; and (c) prolonged blockade of p38 MAPK diminishes tumor growth within the bone. These data indicate that components of cancer-induced bone pain that may reflect ongoing pain and evoked hypersensitivity are likely driven by independent mechanisms. These findings suggest that tumor-induced ongoing pain is dependent on p38 MAPK signaling. However, inhibition of p38 MAPK signaling is insufficient to block hypersensitivity to evoked stimuli, which may be dependent on establishment of altered central processing (i.e., central sensitization). Importantly, we demonstrate that chronic inhibition of p38 MAPK failed to attenuate tactile hypersensitivity despite markedly diminished tumor within the bone and bone remodeling, suggesting potential distinction between anti-tumor activity and tactile hypersensitivity.

Previous studies in mice have demonstrated increased phosphorylated p38 MAPK in the DRG and spinal cord dorsal horn neurons ipsilateral to osteosarcoma treated femurs [27] and in spinal microglia [28], indicating potential peripheral and central actions of p38 MAPK in cancer-induced bone pain. Activation of p38 MAPK has been implicated in numerous translational and transcriptional responses implicated in peripheral and spinal sensitization initiated by inflammatory and neuropathic injury [16,17,29-32]. Previous studies have demonstrated that p38 MAPK mediates increased transcription of nociceptive peptides, such as SP and CGRP, within the DRG [29,33]. Further, p38 MAPK mediates injury-induced increased production of TRPV1 and TRPA1 within the DRG [30,34], and trafficking of the TRPV1 channel to the periphery [30,35,36]. Notably, p38 MAPK drives post-translational changes implicated in pain such as phosphorylation of the TRPV1 channel, resulting in reduced activation threshold and potentiated capsaicin- or proton-evoked responses [37]. Phosphorylation of the Nav1.8 channel increases current density that can increase neuronal excitability under pathological conditions [38]. Thus, the effects of acute administration of SB 203580 may be from inhibition of the direct activation/phosphorylation of pro-nociceptive channels. As tumor- and osteoclast- induced acidosis of the tumor microenvironment are implicated in tumor-induced bone pain [39], increases in acid sensing channels within the bone, decreased activation thresholds within the tumor microenvironment, and increased neuronal excitability, likely result in increased activation of sensory fibers by factors within the tumor microenvironment. Supporting this, blockade of the TRPV1 receptor attenuated osteosarcoma-induced flinching and guarding behavior [39-42]. Thus, prolonged administration of the p38 MAPK inhibitor may diminish cancer-induced ongoing pain behaviors by multiple mechanisms associated with up-regulation and trafficking of pronociceptive signaling channels (e.g., TRPV1) to the periphery.

Within the tumor microenvironment, multiple disease related factors sensitize and directly drive nociceptive afferent fibers [3,43]. In addition to tumor and osteoclast induced acidosis, mechanical factors, such as compression and damage to sensory fibers innervating the bone by tumor growth, or mechanical stress from destabilization of the bone structure due to tumor induced bone remodeling may drive afferent activity [3,43]. Such signal may be amplified due to the phosphorylation of sodium channels [38]. Moreover, pronociceptive inflammatory mediators including endothelins, ATP, prostaglandins, growth factors and pronociceptive cytokines are released from the tumor, tumor-induced bone resorption, and infiltrating immune cells such as tumor associated macrophages [3,27,44-48]. These factors are implicated in sensitization and direct activation of sensory fibers [3,27,44]. Moreover, p38MAPK has been implicated in release of many of these factors including release of ATP from tumor associated macrophages [44] and release of cytokines [22,23,49,50]. More recently, systemic administration of a selective p38α MAPK inhibitor has been shown to inhibit lipopolysaccharide (LPS)-induced elevation of blood levels of tumor necrosis factor-alpha (TNFα) in humans [51]. Thus, the blockade of tumor-induced flinching and guarding behaviors may be due to diminished release of factors within the tumor microenvironment that sensitize primary afferent fibers, thereby lowering thresholds for direct activation by factors within the bone microenvironment (e.g. acid, ATP), and directly reducing factors (e.g. ATP) that may directly drive afferent input from the tumor microenvironment.

In addition to directly inhibiting release of pronociceptive factors from the tumor and associated immune cells, administration of the p38 MAPK inhibitor across 7 days also demonstrated disease modifying effects. Painful bone metastases in humans, such as prostate and breast cancer, often have osteolytic and osteoblastic lesions [52,53]. Our model shows tumor-induced osteolytic lesions by day 10 followed by osteoblastic activity. Prolonged SB203580 treatment completely eliminated the abnormal osteoblastic structures observed in the vehicle-treated bones by day 13 post-tumor implantation. In addition, radiographic analyses of bones showed reduced incidences of spontaneous bone fractures in drug treated bones as compared to those treated with the vehicle. Thus, blockade of tumor-induced bone remodeling likely contributed to the blockade of pain behaviors in cancer bearing mice treated with prolonged administration of the p38 MAPK inhibitor. In addition, histology of bones collected at the end our study show significant tumor reduction with prolonged drug treatment. Moreover, administration of the p38 MAPK inhibitor to cultured 66.1 cells diminished viability, indicating a role of p38 MAPK in tumor growth. This is consistent with multiple reports implicating p38 MAPK in proliferation, invasion and migration of a variety of malignancies including breast cancer [54-58]. These tumorigenic effects of p38 MAPK may be mediated by modulation of transcription factors (e.g. NF-κB [59,60]) and regulation of anti-apoptotic inflammatory signals (e.g. interleukin-6 (IL-6) [61]). SB203580 has been demonstrated to inhibit cyclooxygenase-1 and -2 (COX-1 and 2) [62]. This is notable as COX-2 inhibitors have been demonstrated to diminish tumor growth within bone, bone loss and bone pain in a mouse model of sarcoma-induced bone pain [63]. We demonstrate that treatment with 15 mg/kg of SB203580 restricted tumor growth to the shaft of the bone, with absence of tumor growth into the epiphyseal plate region. At a higher dose, 30 mg/kg, only small pockets of tumor were seen. We note that this is in contrast with previous reports that administration of a p38 MAPK inhibitor across 9-11 days failed to inhibit growth of osteosarcoma cells within the mouse femur [27]. These discrepant results may be due to numerous factors including route of administration (food chow as opposed to twice daily i.p. injections), and cell lines used (osteosarcoma vs. murine cell line 66.1 mammary adenocarcinoma cells). Our data suggest that diminished tumor burden and tumor-induced bone remodeling may be key factors contributing to blockade of tumor-induced flinching and guarding pain behaviors.

Consistent with previous findings in the osteosarcoma pain model [27], p38 MAPK inhibition failed to block tactile hypersensitivity. One potential explanation for the divergent effects of p38 MAPK inhibition on flinching and guarding as opposed to tactile hypersensitivity is the site of action. We propose that activation of afferent fibers innervating the tumor microenvironment drives cancer-induced ongoing pain whereas central mechanisms mediate tactile hypersensitivity. Tactile hypersensitivity is a measure of referred pain, which likely reflects hypersensitivity to touch stimuli, which is mediated by central sensitization [36]. Previous studies have demonstrated phosphorylation of p38 MAPK in the spinal cord across several injury states such as inflammation-induced pain [17,64] neuropathic pain [16,31,65], spinal injury [66] incision [67], and osteosarcoma induced bone pain [27,28]. Several studies have demonstrated that spinal administration of the p38 MAPK inhibitor starting before or at the time of injury blocks evoked pain (tactile and thermal hypersensitivity) [18,68-70]. In contrast, others have demonstrated that spinal administration of p38 MAPK inhibitors after the pain state is established failed to reverse thermal or tactile hypersensitivity [68,71,72]. Thus, although p38 MAPK is implicated in initiation of spinal sensitization, it does not appear to be a factor in maintaining central sensitization and the associated thermal or tactile hypersensitivity. We propose that growth of the tumor within the bone and the associated afferent drive may have established central sensitization required to maintain tactile hypersensitivity prior to drug delivery, as mice demonstrated flinching, guarding, impaired limb use, and tactile allodynia within 7 days, prior to drug administration [36]. Therefore, prolonged and acute administration of the p38 MAPK inhibitor blocked apparent spontaneous pain, but was not sufficient to block the tumor-induced tactile hypersensitivity. Notably, our findings are consistent with a recent clinical report in which systemic administration of a p38 MAPK inhibitor (dilmapimod) to humans with neuropathic pain diminished pain intensity compared to placebo, but failed to alter measures of evoked pain, including tactile allodynia and altered thermal thresholds [73].

## Conclusion

These findings indicate important differences in mechanisms mediating components of bone cancer pain. The observation that the p38 MAPK diminished tumor within the bone indicates that apparent spontaneous pain, as measured by flinching and guarding behaviors, may be dependent on continuing peripheral drive from the tumor whereas central mechanisms established prior to diminished tumor burden maintain evoked pain behaviors. Importantly, this may indicate different mechanisms might drive ongoing pain as opposed to evoked pain. A better understanding of how mechanisms driving multiple components of bone cancer pain may lead to a more complete therapeutic treatment strategy.

## Methods

### Murine cell line: 66.1

Murine cell line 66.1 derived from spontaneously occurring mammary adenocarcinoma was maintained in advanced Minimum Essesntial Medium, Eagle (αMEM) (Cellgro Mediatech) containing 10% Fetal bovine serum (Gemini Bioproducts), 100 units/mL penicillin, and 100 units/mL streptomycin at 37°C and in a 5% CO_2 _atmosphere. The cells were passaged every 3 days, and harvested between 12 and 21 passages.

### Mouse strain

Female adult Balb/cfC3H mice, weighing 20-25 g (Jackson Laboratories, Bar Harbor, ME), were chosen for histocompatability with the 66.1 cells. All mice were housed maximum three per cage and used in strict accordance with the NIH Health Guide for the Care and Use of Laboratory Animals and the University of Arizona Animal Care UnitSurgery

### Surgery

Baseline radiograph images (Faxitron X-ray Corporation) of the right femurs were obtained prior to surgery. Animals were anesthetized with ketamine (80 mg/kg)/xylazine (12 mg/kg) i.p. and an arthrotomy was performed exposing the condyles of the distal femur as previously described [74]. A hole was drilled into the femur for injecting the needle. Needle placement inside the intramedullary space of the femur was verified using radiograph images and 5 μl of serum-free minimal essential medium (MEM) containing approximately 5 × 10^5 ^cells was injected into the intramedullary space of the right femur. For cell-free controls, 5 μl of serum-free MEM alone (no cells) was injected and sealed into the femur. The injection site was sealed with dental cement (Simplex).

### Drug

SB203580-hydrochloride (Tocris # 1402) (15 mg/kg and 30 mg/kg, i.p.), a selective inhibitor of the activity of p38 MAPK, was dissolved in saline on a heat stir plate at 40°C in a flask covered with aluminum foil. To determine the effects of acute administration of SB203580, the drug was administered 13 days following the injection of 66.1 cells into the femur. To determine the effects of chronic drug administration, it was administered at two doses (15 mg/kg and 30 mg/kg, i.p. 2× daily across 7 days starting day 7 post surgery). The animals were divided into four groups: Cancer-SB203580, Cancer-saline, Control-SB203580 and control-saline with 10-12 animals in each group.

## Behavioral Analysis

### Experimental Design

Each mouse was tested for movement-evoked pain (limb use), spontaneous pain behaviors (flinching and guarding), and tactile allodynia prior to surgery (pre-sugery baselines). To determine the pain behavior following the injection of breast cancer cells into the femur, mice were tested on days 7, 9, 11 and 13 post-surgery. Acute effects of SB203580 were tested on day 13 post surgery. Pre-drug analysis of spontaneous pain and tactile allodynia were obtained for all groups, followed by drug administration. Behaviors were again assessed at 30, 60, 90, 120 and 180 min following drug administration. All behaviors were assessed within the same animal. Each animal was first tested for flinching and guarding followed by tactile allodynia. To avoid repeated handling and transferring of animals from testing chambers to the walking pan at each time point separated by 30 min for the acute drug studies, limb use scoring was only carried out for the chronic administration of SB203580. To study the chronic effects of SB203580 administration, animals were tested for movement-evoked pain (limb use), spontaneous pain behaviors (flinching and guarding), and tactile allodynia prior to surgery (pre-surgery baselines) following the surgery on days 7 (pre-drug), 9, 11 and 13. The behavioral tests were carried out at time points later than observed peak effect of acute administration of the drug. Mice were first tested for limb use by allowing them to walk in an empty mouse pan and then placed in testing chambers for assessment of spontaneous pain and tactile allodynia. All pain measures were conducted by the experimenter who was blinded to the treatments.

### Spontaneous pain behaviors

All mice were placed in suspended plexiglass chambers with a wire grid floor and allowed to acclimate to the chamber for 1 h. Guarding and flinching behaviors were measured during a 2 min observation period. The number of flinching episodes and the total time spent guarding the foot of the cancer bearing limb were measured across 2 minutes for each mouse.

### Limb Use

Limb use was assessed as previously described [74]. The mouse was placed in an empty mouse pan and observed while walking across the pan in a continuous motion. Limping and/or guarding behavior of the right (cancer treated) hind-limb was rated on the following scale: 0 = complete lack of use, 1 = partial non-use, 2 = limping and guarding, 3 = limping, 4 = normal walking.

### Tactile hypersensitivity

Paw withdrawal thresholds in response to probing with calibrated von Frey filaments were determined using the "up-down" method described by Chaplan et al [75]. Mice were kept in suspended cages with wire grid floors and the von Frey filament applied perpendicularly to the plantar surface of the ipsilateral paw until it buckled. A positive response was indicated by a sharp withdrawal of the paw. An initial probe equivalent to 2 g was applied and if the response was negative, the stimulus was incrementally increased until a positive response was obtained, then decreased until a negative result was obtained. This up-down method was repeated until three changes in behavior were determined, and the pattern of positive and negative responses was tabulated. The 50% paw withdrawal threshold was determined as previously described [76].

## Determination of bone destruction and tumor burden

### Radiographical Analysis

To determine the effects of chronic administration of SB203580, radiographs were taken after behavioral testing on post-surgery days 7 (pre-drug) and 13 using a Faxitron machine. Images were captured by a digital camera. Bone loss was rated by an experimenter blinded to treatment according to a 5-point scale: 5 = normal bone 4 = epiphyseal bone loss, 3 = enhanced bone loss, 2 = osteoblastic structures 1 = unicortical fractures, 0 = bicortical fractures.

### Bone histology

On day 13, following the behavioral testing and radiograph imaging, all mice received an overdose of ketamine (80 mg/kg)/xylazine (12 mg/kg) i.p. and were perfused transcardially with saline followed by 10% neutral-buffered formalin (Sigma, St. Louis, MO, USA). Femurs were collected from the right (cancer-treated) leg and postfixed overnight in 10% neutral buffered formalin. Femurs were rinsed in water to remove formalin and then placed in Decal solution (RDO-Apex, Aurora, IL), for an hour to achieve decalcification. Samples were dehydrated in graded ethanol solution before carefully embedding in paraffin. They were oriented so that the entire length of the femur could be longitudinally sectioned. Sections 3 μm thick were stained with hematoxylin and eosin to visualize normal marrow elements and cancer cells under bright field microscopy. The specimens were observed using an Olympus microscope and images were collected using an Olympus color camera with proprietary software. For image analysis, bone histology images were analyzed using image J (NIH). Area occupied by tumor was presented as percent of the total area of the intramedullary space within the bone, calculated as: (tumor area/total area)*100.

## In vitro analysis of breast cancer cell growth

The 66.1 cells were maintained in MEM media as mentioned above. To evaluate the effect of SB203580 on tumor cell growth, 66.1 cells were treated with 10 or 20 μM of SB203580. Cells treated with 4% DMSO grown at the same time served as controls. Cells were seeded at 15000 cells/well in 6-well plates in complete media for 36 h. When cells reached the confluency of 30-50%, they were serum starved overnight with serum free optiMEM media. After 48 h of drug treatment, cell growth was arrested by slowly adding 50% Tri-chloro acetate (TCA) and the cells were kept at 4°C for a minimum of 1 h. All cells were then washed with distilled water 4-5 times and then treated with 1 ml of sulforhodamine-B (SRB) dye for 10-15 min at room temperature. The cells were again washed with distilled water 4-5 times. They were then precipitated in 0.1% acetic acid on a shaker for 10 min. The absorbance was read at 540 nm. For data analysis, the mean OD_540 _in the SB203580-treated cells is compared to that of the control done in parallel. Normalized data from three independent experiments are expressed as means ± SEM.

## Statistical Analysis

Statistical comparisons between treatment groups were done using one or two way ANOVA. Post-hoc comparisons between groups were done using Bonferroni posthoc test. To determine the correlation between tumor-induced bone remodeling and tumor-induced pain behaviors, linear regression analysis was carried out. No correlation was found between bone remodeling and pain behaviors (R < 0.35, p < 0.05). Therefore, data for all animals were analyzed together. For all analysis, significance was set at *p *< 0.05.

## List of Abbreviations

MAPK: mitogen activated protein kinase; SP:substance P; CGRP: calcitonin gene related peptide; TNF: tumor necrosis factor; IL: Interleukin; ip: intraperitoneal

## Competing interests

The authors declare that they have no competing interests.

## Authors' contributions

DS and AO: Performed the behavioral studies. DS performed the radiographic and molecular analysis. DS, AC, MN: performed the tissue culture and cell viability analysis. DS, MN, TV, FP and TK: participated in the design of the study. DS, TK and FP: wrote and edited the manuscript. All authors read and approved the final manuscript.
